# Risk Factors for Bleeding Varicose Veins in Patients with Chronic Venous Disease

**DOI:** 10.3390/medicina59061034

**Published:** 2023-05-27

**Authors:** Davide Costa, Nicola Ielapi, Roberto Minici, Antonio Peluso, Umberto Marcello Bracale, Michele Andreucci, Raffaele Serra

**Affiliations:** 1 Department of Law, Economics and Sociology, University “Magna Graecia” of Catanzaro, 88100 Catanzaro, Italy; davide.costa@unicz.it; 2 Interuniversity Center of Phlebolymphology (CIFL), International Research and Educational Program in Clinical and Experimental Biotechnology, University “Magna Graecia” of Catanzaro, 88100 Catanzaro, Italy; nicola.ielapi@uniroma1.it; 3 Department of Public Health and Infectious Disease, “Sapienza” University of Rome, 00185 Rome, Italy; 4 Department of Experimental and Clinical Medicine, University “Magna Graecia” of Catanzaro, 88100 Catanzaro, Italy; miniciroberto@gmail.com; 5 Department of Public Health, University of Naples “Federico II”, 80138 Naples, Italy; antoniopeluso8784@gmail.com (A.P.); umbertomarcello.bracale@unina.it (U.M.B.); 6 Department of Health Sciences, University “Magna Graecia” of Catanzaro, 88100 Catanzaro, Italy; andreucci@unicz.it; 7 Department of Medical and Surgical Sciences, University “Magna Graecia” of Catanzaro, 88100 Catanzaro, Italy

**Keywords:** chronic venous disease, varicose veins, bleeding, hemorrhage, risk factors

## Abstract

*Background and Objectives*: Chronic venous disease (CVD) is a widespread clinical condition that is very common in western countries in the adult general population with a wide range of clinical manifestations, such as varicose veins (VVs) that in certain circumstances may complicate with rupture and subsequent bleeding that may even be fatal. The aim of this study is to evaluate risk factors for bleeding VVs. *Materials and Methods*: This is a retrospective study conducted in patients with CVD complicating with bleeding of VVs over a 4-year period (2019–2022). A random sample, for the same 4-year period and with a 3:1 ratio, was selected from other CVD patients without VVs bleeding that served as the control group. *Results*: From a global population of 1048 patients with CVD over a 4-year period, a total of 33 patients (3.15%) with VVs bleeding were selected. A group of 99 patients without VVs bleeding were randomly selected from the total population of 1048 patients with CVD. Findings of this study showed that advanced clinical stage of CVD (i.e., C4b stage), advanced age, living alone, suffering from cardiovascular co-morbidity (i.e., hypertension and CHF), assuming certain drugs that act on blood coagulation (i.e., aspirin, anticoagulants), assuming psychotropic medication, having particular venous reflux patterns (i.e., below-knee GSV reflux, non-saphenous veins reflux, Cockett’s perforators reflux), and not having been assessed and treated previously for CVD (i.e., with VADs, CT, or surgery) may predispose a high risk for bleeding VVs. *Conclusions*: Bleeding VVs may be a life-threatening complications of CVD patients, and monitoring risk factors found in this study and others that, hopefully, may be discovered in the future through further focused research will help to reduce the impact of this problem in this patient population.

## 1. Introduction

Chronic venous disease (CVD) is a widespread clinical condition that is very common in western countries with a prevalence up to 57% and 77% in men and women, respectively, in the adult general population. It is a clinical condition characterized by functional and morphological changes that may occur in the venous system of the lower limbs and by a wide range of clinical manifestations that also impair social determinants of health and is, subsequently, associated with a reduced quality of life [1,2]. Varicose veins (VVs), one of the most common signs of CVD, are ectatic, tortuous veins of the superficial venous system that are at least 3 mm in size and are also characterized by architectural and structural alterations of the vein wall [3,4]. VVs are not always as benign as they are often perceived; among several complications that may occur in varicose veins, bleeding, although rare, is the most dreadful one because it may be potentially fatal [5]. In fact, 1 out of 1000 autopsies for sudden death are due to bleeding for ruptured VVs [6]. Nevertheless, in the current literature, there are also some case series describing non-fatal hemorrhage from ruptured VVs [7,8,9]. Overall, the general reported incidence of bleeding VVs ranges from 3% to 9.1% [10].

Due to the rarity of this clinical condition, risk factors predisposing to such complication are not fully understood, and the aim of this paper is to retrospectively study characteristics of patients with CVD complicating with VVs bleeding that referred to our departments.

## 2. Materials and Methods

### 2.1. Study Design

This study is a multicenter (University Magna Graecia of Catanzaro and at University Federico II of Naples, belonging to the Interuniversity Center of Phlebolymphology (CIFL) International Research and Educational Program in Clinical and Experimental Biotechnology) analysis of retrospectively collected data of consecutive patients with CVD referred to our departments for bleeding VVs from January 2019 to December 2022. Inclusion criteria include (I) patients with CVD and a bleeding event of VVs; (II) both sexes; (III) age of at least 18 years. The exclusion criteria include (I) bleeding not related to VVs or CVD.

Specifically, a random sample with a 3:1 ratio was selected from other CVD patients without VVs bleeding that served as the control group.

The study was approved by the Institutional Review Board of Interuniversity Center of Phlebo-lymphology (CIFL) International Research and Educational Program in Clinical and Experimental Biotechnology (Approval number: ER.ALL.2018.63A).

### 2.2. Data Collection

Data were obtained from medical records that were reviewed for patients’ characteristics from both groups and were also classified according to the clinical stages (C) of the (Clinical-Etiology-Anatomy-Pathophysiology) CEAP classification [11].

In particular, the following data were collected: age, gender, family members, body mass index (BMI), and smoking.

The following pre-existing diseases were collected: hypertension, diabetes, dyslipidemia, coronary artery disease (CAD), congestive heart failure (CHF), chronic kidney disease (CKD), chronic obstructive pulmonary disease (COPD), stroke, previous superficial vein thrombosis (SVT), previous deep vein thrombosis (DVT), and post thrombotic syndrome (PTS).

The following pharmacological and surgical treatments were collected: aspirin, anticoagulation, statin, antihypertensive, diabetes medication, psychotropic medication, veno-active drugs (VADs), compression therapy, previous open surgery, previous endovascular surgery, and previous wound care.

### 2.3. Data Analysis

The 3:1 ratio randomization to obtain the control group was performed with random number generators (https://www.calculatorsoup.com/calculators/statistics/random-number-generator.php) (last accessed on 18 April 2023).

Data were maintained in an Excel spreadsheet (version 16.72—Microsoft Inc., Redmond, DC, USA). The statistical analysis was carried out with RStudio (version 1.4.1106, 2009–2021, PBC). Continuous variables (age, family members) were analyzed with a Welch Two Sample *t*-test and presented as mean ± standard deviation. The remaining categorical variables were subjected to two-sample test for equality of proportions with continuity correction and showed as frequency (percentage value).

All significant categorical variables were also analyzed with Fisher’s test to quantify the statistical association across odds ratio and 95% confident interval values.

## 3. Results

From a global population of 1048 patients with CVD over a 4-year period, a total of 33 patients (3.15%) with VVs bleeding were selected.

A group of 99 patients without VVs bleeding were randomly selected from the total population of 1048 patients with CVD.

Non-bleeding patients are labeled as Group A, and bleeding patients are labeled as Group B for statistical analysis.

The mean age of the two groups A and B (*n* = 132) is 49.2 ± 14 years while the mean age of group B is equal to 61.7 ± 10.6. Regarding gender in general, 65.9% are women while in group B, 54.5% are women. Group B has fewer family members than group A (3.5 ± 1.2 vs. 1.2 ± 0.5).

Demographics of study population are shown in Table 1.

All the patients presenting with bleeding VVs had reported bleeding from one lower limb only, and there was no patient who had bleeding from the bilateral limbs on presentation. There was no fatal bleeding. All the patients with bleeding VVs were treated with open surgery venous ablation (phlebectomy).

Clinical and hemodynamic characteristics are shown in Table 2.

Among the variables considered, some were particularly significant. Regarding co-morbidities, the following were found: hypertension (*p*-value < 0.001), CHF (*p*-value = 0.013) previous SVT (*p*-value = 0.007), and previous DVT (*p*-value = 0.013).

Among pharmacological and surgical treatments were the following: aspirin (*p*-value < 0.001), anticoagulation (*p*-value < 0.001), psychotropic medication (*p*-value = 0.007), previous open surgery (*p*-value = 0.013), VADs (*p*-value < 0.001), and CT (*p*-value < 0.001).

Significant results were found for clinical and hemodynamics characteristics: SFJ throughout entire GSV reflux (*p*-value = 0.010), below-knee GSV reflux (*p*-value = 0.006), non-saphenous vein reflux (*p*-value < 0.001), CEAP class C2 (*p*-value < 0.001), CEAP class C4b (*p*-value = 0.010), and Cockett perforators reflux (*p*-value < 0.001).

To analyze quantification of the association for significant variables, Fisher’s test was performed as shown in Table 3.

Overall, statistical analysis highlighted the significant association between bleeding VVs and more advanced age, low number of family members, comorbidities such as hypertension, CHF, previous SVT, previous DVT, assumption of aspirin and anticoagulants, assumption of psychotropic medication, the presence of more advanced CVD such as skin changes, and hemodynamic abnormalities such as below-knee great saphenous (GSV) reflux, non-saphenous veins reflux, and Cockett’s perforators reflux.

Protective factors resulted in the following: assumption of VADs, the use of CT such as elastic stockings, and previous surgical and/or endovascular surgery for VVs and hemodynamic abnormalities such as sapheno-femoral junction (SFJ) throughout entire GSV reflux, and mild venous disease symptoms (C2 of CEAP classification).

Considering clinical presentation, all 33 patients with bleeding presented with moderate severity of bleeding, with non-critical hemoglobin levels. In 23 cases (69.70%), minor local accidental trauma was reported; in 7 patients (21.21%), no apparent cause was reported; and in 3 cases (9.09%), the bleeding originated from ulcerated skin.

## 4. Discussion

The main sites of superficial venous system of lower limbs that can be involved in CVD pathophysiology and vein reflux are represented by GSV, small saphenous veins (SSV), anterior accessory saphenous veins (AASVs), SFJ, sapheno-popliteal junction (SPJ), perforators veins, and non-saphenous veins [12]. In particular, the superficial veins connect to the deep veins at the SFJ, where the GSV joins the common femoral vein, and at the SPJ where the SSV joins the popliteal vein [13]. Additionally, several perforator veins, such as Dodd’s, Boyd’s, and Cockett’s perforators, connect the deep and the superficial venous system along the lower limb and may be responsible of important vein reflux [14].

Vein reflux, in one or more of the aforementioned veins, is the fundamental mechanisms in CVD and is responsible of the formation of bulging VVs, visible on lower limbs’ skin, and of more important signs such as skin changes, leg ulceration, and even bleeding VVs [13]. Reflux is also responsible of local venous hypertension that determines venous shear stress on the endothelium, which triggers biochemical and cellular events that stimulate inflammation pathways that characterize all stages and complication of CVD [15,16,17].

Hemorrhage from VVs is a dreadful event that presupposes a series of concurrent factors not yet fully understood in CVD patients [6].

In this study, 3.15% of patients with CVD were found to have bleeding complication. As our centers do not have dedicated emergency units, patients with VVs bleeding were directed to our departments from external emergency centers or directly by general practitioners. Thus, the figure of 3.15% probably underestimates the real number of patients suffering from this venous complication. Generally, the overall incidence in the current literature is underestimated as patients had often suffered from multiple minor bleeds before the bleed that led to referral, and considering that most of those minor bleeds are managed outside the hospital setting, they are frequently not referred or registered [10].

In this study, several factors resulted as significantly associated with the risk of bleeding VVs. In particular, some demographic elements include increasing age and living alone; advanced CVD stages, such as severe skin changes (C4b of CEAP classification); some comorbidities such as hypertension, CHF, or previous acute disease of the venous system such as SVT and DVT; some drug treatments such as anticoagulants and/or aspirin assumption and psychotropic medications; some venous hemodynamic factors such as below-knee great saphenous (GSV) reflux, non-saphenous veins reflux, and Cockett’s perforators reflux. SFJ throughout entire GSV reflux seems to even protect affected patients from bleeding VVs. The role of classical treatments, such as VADs, CT, and surgery (open and/or endovascular) clearly represent protective strategies against the possibility of bleeding VVS.

From a hemodynamic point of view, it is evident that venous hypertension may play a pivotal role in determining the progressive weakness of the vein wall till rupture. In fact, perforator veins reflux has been found in more than 70% of patients with severe CVD and represents a known risk factor for severe CVD. Perforator veins reflux is able to generate high pressure from deep veins through volume overload at connection points of superficial veins to which they are connected with subsequent incompetence of the superficial veins, but they may further overload previously incompetent superficial veins affecting their re-entry points [18]. Furthermore, in patients with skin changes and/or venous leg ulceration (VLU) that occur in the most severe clinical stage of CVD (C4-C6 of CEAP classification), quite often it is possible to find isolated below-knee GSV reflux and/or perforator veins reflux without the presence of above-knee GSV reflux. These conditions are obviously characterized by high venous pressures in the distal part of the lower limbs and are in accordance with the ascending theory where the pathological events of CVD are supposed to start in the distal superficial veins of the leg extending proximally [19,20]. These hemodynamic conditions have also been found to be significantly associated in the bleeding VVs group of CVD patients of this study.

Furthermore, non-saphenous veins reflux was particularly associated with bleeding VVs of this study. A sort of private circulation with local recirculation with important extra saphenous overload [21] may determine particular local high pressure resulting in vein wall damage that may predispose to rupture and bleeding.

Previous SVT and DVT are often associated with development of superficial and perforators vein reflux in most affected limbs and sometimes also of skin complications during SPT and can be easily considered as risk factors for bleeding VVs [6,22].

Increasing age is characterized by more advanced stages of CVD and by more fragile skin, and this can further elevate the risk of VVs bleeding. In particular, skin over long-lasting varicose veins may get thinner over time, and bluish dark veins can even be observed through the fragile skin [6,23].

Clinically, varicose hemorrhage may be spontaneous or may occur in a context of minor, quite unremarkable trauma (i.e., accidental scratching of a VV, even by pets’ scratch, bumping leg against the furniture, leg shaving, etc.) Very often, the bleeding occurs in a subject in erect posture—for example, standing while taking a shower—as the further increase in venous blood pressure, in a context of a frank venous hypertension, the extra wall tension in a weak area with vein wall and skin damage may trigger the acute rupture of VVs. Moreover, bleeding VVs may also occur during or directly after encountering warm water—for example, during washing—due to heat-induced peripheral vasodilation [6,24].

Another interesting demographic factor found in this study regards the relation between the risk of bleeding VVs and the low number of family members living with the patient. As demonstrated in the current literature, patients’ families also function as care givers, especially for elderly family members with social, psychological, emotional, and financial support [25]. Taking into consideration the role of families and patients, it is evident how family relationships affect therapeutic decisions and, above all, the prevention of bleeding risk [26]. Furthermore, involvement in strong supportive families is consistently associated with better health outcomes. Conversely, living in conditions of social isolation determines worse health outcomes in patients [27,28], such as the risk for bleeding in CVD patients.

Moreover, elderly subjects living alone, in case of bleeding VVs, may miss the chance to receive immediate correct first aid assistance and may be not able to apply correct hemostasis control techniques, thus resulting in fatal events, and it should also be considered that if the venous bleeding is massive, the patient’s death can occur in a few minutes [6].

The correlation of CHF and hypertension does not seem to rely, according to the actual knowledge, with vein pathophysiologic events, but it seems related to the general poor health outcomes of affected patients [6,29].

In this study, the assumption of aspirin and anticoagulants has been significantly related to bleeding VVs, and this is consistent with the blood anticoagulative modifications inducted by these drugs. In this context, Maher et al. reported a case of spontaneous bleeding of VVs into the posterior superficial compartment of the calf in a patient receiving enoxaparin, aspirin, and clopidogrel [30].

CVD patients requiring the assumption of psychotropic medication may have mental problems that further affect appropriate care for their legs and generally affect self-preservation [6,31]. On the other hand, appropriately caring clinical manifestation of CVD with current treatments (VADs, CT, open and endovascular surgery) results in good prevention of bleeding VVs as showed by the findings of this study.

If timely recognized, bleeding VVs can be effectively treated using open and/or endovascular procedures [9]. Therefore, it is important that all patients with bleeding VVs should be referred immediately to vascular surgery services [10].

The most important point of this article is that the risk factors evaluated in this study can define subgroups of CVD patients that are at a particular increased risk of bleeding complication—in particular, patients with anticoagulation, living alone, having non-saphenous veins reflux and/or expansion of lower limb perforator veins (specifically Cockett’s perforator veins), impaired mental conditions, and, most of all, not having compression therapy. Moreover, among risk factors for bleeding found in this study, the only definitely modifiable one able to prevent severe complications of bleeding from VVs is regular compression therapy, which is one of the mainstays of CVD treatment [1].

Limitations of the study are mainly represented by the accuracy of the medical records coming from routine clinical practice collected general data without a specific focus, and a great deal of important data were probably missed; furthermore, because the patients’ series were uncontrolled.

## 5. Conclusions

This study showed that advanced clinical stage of CVD (i.e., C4b stage), advanced age, living alone, suffering from cardiovascular co-morbidity (i.e., hypertension and CHF), assuming certain drugs that act on blood coagulation (i.e., aspirin, anticoagulants), assuming psychotropic medication, having particular venous reflux patterns (i.e., below-knee GSV reflux, non-saphenous veins reflux, Cockett’s perforators reflux), and not having been assessed and treated previously for CVD (i.e., with VADs, CT, or surgery) may predispose a high risk for bleeding VVs. Further research is needed in this area to better identify prevention strategies for the risk of this life-threatening complication in patients affected by VVs.

## Figures and Tables

**Table 1:** Demographics and clinical characteristics of study population.

	Overall (N = 132)	Group A (No Bleeding) *n* = 99	Group B (Bleeding) *n* = 33	CI95%	*p*-Value (<0.05)
**Subject clinical characteristics**					
**Age** (years)	49.2 ± 14	45 ± 12.5	61.7 ± 10.6	[−21.1; −12.2]	<0.001
**Females**	87 (65.9%)	69 (69.6%)	18 (54.5%)	[−0.06; 0.36]	0.168
**BMI** (kg/m^2^)	25.4 ± 4.1	25.1 ± 4.1	26.2 ± 3.8	[−2.68; 0.47]	0.166
**Current smoker**	45 (34.1%)	34 (34.3%)	11 (33.3%)	[−0.18; 0.20]	1.000
**Hypertension** (SAP > 140 mmHg and/or DAP > 90 mmHg)	18 (13.6%)	6 (6%)	12 (36.3%)	[−0.49; −0.11]	<0.001
**Diabetes** (glycemia > 125 mg/dL and/or use of HD/insulin)	15 (11.3%)	10 (10.1%)	5 (15.1%)	[−0.20; 0.10]	0.634
**Dyslipidemia** (Tot. Chol. > 240 mg/dL and/or TGL > 150 mg/dL and/or use of LLD)	11 (8.3%)	5 (5.1%)	6 (18.1%)	[−0.28; 0.02]	0.045
**CAD**	6 (4.5%)	2 (2%)	4 (12.1%)	[−0.23; 0.03]	0.053
**CHF**	5 (3.7%)	1 (1%)	4 (12.1%)	[−0.24; 0.02]	0.017
**CKD** (GFR < 60 mL/min/1.73 m^2^)	2 (1.5%)	1 (1%)	1 (3%)	[−0.10; 0.06]	1.000
**COPD**	3 (2.2%)	2 (2%)	1 (3%)	[−0.08; 0.06]	1.000
**Stroke**	4 (3%)	1 (1%)	3 (9.1%)	[−0.20; 0.04]	0.078
**Previous SVT**	9 (6.8%)	3 (3%)	6 (18.1%)	[−0.30; 0.004]	0.009
**Previous DVT**	5 (3.7%)	1 (1%)	4 (12.1%)	[−0.24; 0.02]	0.017
**PTS**	4 (3%)	1 (1%)	3 (9.1%)	[−0.20; 0.04]	0.078
**Aspirin**	14 (10.6%)	4 (4%)	10 (30.3%)	[−0.44; −0.08]	<0.001
**Anticoagulation**	9 (6.8%)	2 (2%)	7 (21.2%)	[−0.35; −0.02]	<0.001
**Statin**	11 (8.3%)	5 (5.1%)	6 (18.1%)	[−0.28; −0.02]	0.045
**Antihypertensive**	18 (13.6%)	6 (6%)	12 (36.3%)	[−0.49; −0.11]	<0.001
**Diabetes medication**	15 (11.3%)	10 (10.1%)	5 (15.1%)	[−0.20; 0.10]	0.634
**Psychotropic medication**	9 (6.8%)	3 (3%)	6 (18.1%)	[−0.30; 0.004]	0.009
**Previous open surgery**	28 (21.2%)	26 (26.2%)	2 (6%)	[0.06; 0.34]	0.026
**Previous endovascular surgery**	7 (5.3%)	6 (6%)	1 (3%)	[−0.06; 0.12]	0.822
**Previous wound care**	3 (2.2%)	2 (2%)	1 (3%)	[−0.08; 0.06]	1.000
**VADs**	83 (62.8%)	80 (80.8%)	3 (9.1%)	[0.57; 0.86]	<0.001
**CT**	78 (59.1%)	76 (76.7%)	2 (6%)	[0.57; 0.84]	<0.001
**Family members**	2.9 ± 1.4	3.5 ± 1.2	1.2 ± 0.5	[1.98; 2.60]	<0.001

BMI = body mass index; SAP = systolic arterial blood pressure; DAP = diastolic arterial blood pressure; HD = hypoglycemic drugs; TGL = triglycerides; LLD = lipid-lowering-drugs; CAD = coronary artery disease; CHF = congestive heart failure; CKD = chronic kidney disease; COPD = chronic obstructive pulmonary disease; GFR = glomerular filtration rate; SVT = superficial vein thrombosis; DVT = deep vein thrombosis; PTS = post thrombotic syndrome; VADs = veno-active drugs; CT = compression therapy.

**Table 2:** Clinical and hemodynamics characteristics.

Clinical and Hemodynamic Characteristics	Overall (N = 132)	Group A (No Bleeding) *n* = 99	Group B (Bleeding) *n* = 33	CI95%	*p*-Value (<0.05)
**CEAP class:**					
- **C2**	104 (78.7%)	85 (85.8%)	19 (57.5%)	[0.08; 0.48]	0.001
- **C3**	12 (9.1%)	8 (8.1%)	4 (12.1%)	[−0.18; 0.10]	0.726
- **C4a**	3 (2.2%)	2 (2%)	1 (3%)	[−0.08; 0.06]	1.000
- **C4b**	7 (5.3%)	2 (2%)	5 (15.1%)	[−0.27; 0.01]	0.013
- **C4c**	1 (0.7%)	0 (0%)	1 (3%)	[−0.10; 0.04]	0.562
- **C6**	5 (3.7%)	2 (2%)	3 (9.1%)	[−0.19; 0.05]	0.188
**SFJ throughout entire GSV reflux**	45 (34.1%)	40 (40.4%)	5 (15.1%)	[0.07; 0.42]	0.014
**Anterior accessory saphenous vein reflux**	13 (9.8%)	11 (11.1%)	2 (6%)	[−0.07; 0.17]	0.612
**SFJ and GSV reflux above the knee**	7 (5.3%)	5 (5.1%)	2 (6%)	[−0.11; 0.09]	1.000
**Below-knee GSV reflux**	14 (10.6%)	6 (6%)	8 (24.2%)	[−0.35; −0.008]	0.009
**Segmental GSV reflux**	14 (10.6%)	10 (10.1%)	4 (12.1%)	[−0.16; 0.12]	1.000
**GSV reflux not involving the SFJ**	11 (8.3%)	9 (9.1%)	2 (6%)	[−0.08; 0.14]	0.855
**SFJ reflux only**	5 (3.7%)	3 (3%)	2 (6%)	[−0.13; 0.07]	0.792
**Non-saphenous veins reflux**	31 (23.3%)	6 (6%)	25 (75.7%)	[−0.87; −0.52]	<0.001
**Entire SSV reflux**	24 (18%)	16 (16.1%)	8 (24.2%)	[−0.26; 0.10]	0.434
**SPJ reflux only**	3 (2.2%)	2 (2%)	1 (3%)	[−0.08; 0.06]	1.000
**Segmental SSV reflux**	10 (7.5%)	8 (8.1%)	2 (6%)	[−0.09; 0.13]	1.000
**Entire SSV reflux with thigh extension**	10 (7.5%)	7 (7%)	3 (9.1%)	[−0.15; 0.11]	1.000
**Perforators reflux:**					
- **Cockett**	17 (12.8%)	6 (6%)	11 (33.3%)	[−0.46; −0.08]	<0.001
- **Dodd**	3 (2.2%)	1 (1%)	2 (6%)	[−0.15; 0.05]	0.311
- **Boyd**	3 (2.2%)	1 (1%)	2 (6%)	[−0.15; 0.05]	0.311
**Deep venous system reflux**	3 (2.2%)	1 (1%)	2 (6%)	[−0.15; 0.05]	0.311
**Right bleeding limb**	15 (11.3%)	-	15 (45.4%)	-	-
**Site of bleeding:**					
- **Distal thigh**	5 (15.1%)	-	5 (15.1%)	-	-
- **Proximal leg**	3 (9.1%)	-	3 (9.1%)	-	-
- **Distal leg**	3 (9.1%)	-	3 (9.1%)	-	-
- **Ankle**	8 (24.2%)	-	8 (24.2%)	-	-
- **Calf**	9 (27.2%)	-	9 (27.2%)	-	-
- **Foot**	5 (15.1%)	-	5 (15.1%)	-	-

SFJ = saphenofemoral junction; GSV = great saphenous vein; SSV = small saphenous vein; SPJ = saphenopopliteal junction.

**Table 3:** Quantification of the association for significant variables with Fisher’s test.

	Overall (N = 132)	Group A (No Bleeding) *n* = 99	Group B (Bleeding) *n* = 33	Odds Ratio	CI95%	*p*-Value (<0.05)
**Subject clinical characteristics**						
**Hypertension** (SAP > 140 mmHg and/or DAP > 90 mmHg)	18 (13.6%)	6 (6%)	12 (36.3%)	8.6	[2.65; 31.5]	<0.001
**CHF**	5 (3.7%)	1 (1%)	4 (12.1%)	13.1	[1.24; 669.5]	0.013
**Previous SVT**	9 (6.8%)	3 (3%)	6 (18.1%)	6.9	[1.38; 45.9]	0.007
**Previous DVT**	5 (3.7%)	1 (1%)	4 (12.1%)	13.1	[1.24; 669.5]	0.013
**Aspirin**	14 (10.6%)	4 (4%)	10 (30.3%)	10.1	[2.62; 48.1]	<0.001
**Anticoagulation**	9 (6.8%)	2 (2%)	7 (21.2%)	12.7	[2.24; 132.8]	<0.001
**Psychotropic medication**	9 (6.8%)	3 (3%)	6 (18.1%)	6.9	[1.38; 45.9]	0.007
**Previous open surgery**	28 (21.2%)	26 (26.2%)	2 (6%)	0.18	[0.01; 0.80]	0.013
**VADs**	83 (62.8%)	80 (80.8%)	3 (9.1%)	0.02	[0.004; 0.09]	<0.001
**CT**	78 (59.1%)	76 (76.7%)	2 (6%)	0.02	[0.002; 0.08]	<0.001
**Clinical and hemodynamics characteristics**						
**SFJ throughout entire GSV reflux**	45 (34.1%)	40 (40.4%)	5 (15.1%)	0.26	[0.07; 0.77]	0.010
**Below-knee GSV reflux**	14 (10.6%)	6 (6%)	8 (24.2%)	4.8	[1.34; 18.8]	0.006
**Non-saphenous vein reflux**	31 (23.3%)	6 (6%)	25 (75.7%)	45.6	[13.6; 181.3]	<0.001
**CEAP class C2**	104 (78.7%)	85 (85.8%)	19 (57.5%)	0.22	[0.08; 0.60]	0.001
**CEAP class C4b**	7 (5.3%)	2 (2%)	5 (15.1%)	8.4	[1.30; 93.6]	0.010
**Cockett’s perforators reflux**	17 (12.8%)	6 (6%)	11 (33.3%)	7.5	[2.28; 27.9]	<0.001

CHF = congestive heart failure; SVT = superficial vein thrombosis; DVT = deep vein thrombosis; VADs = veno-active drugs; CT = compression therapy; SFJ = saphenofemoral junction; GSV = great saphenous vein.

## Data Availability

All data generated or analyzed during this study are included in this published article.

## Abbreviations

BMI	body mass index
CAD	coronary artery disease
CHF	congestive heart failure
CHF	congestive heart failure
CKD	chronic kidney disease
COPD	chronic obstructive pulmonary disease
CT	compression therapy
CVD	chronic venous disease
DAP	diastolic arterial blood pressure
DVT	deep vein thrombosis
GFR	glomerular filtration rate
GSV	great saphenous vein
HD	hypoglycemic drugs
LLD	lipid-lowering-drugs
PTS	post thrombotic syndrome
SAP	systolic arterial blood pressure
SFJ	saphenofemoral junction
SPJ	saphenopopliteal junction
SSV	small saphenous vein
SVT	superficial vein thrombosis
TGL	triglycerides
VADs	veno-active drugs
VV	varicose vein
